# AgNP-PVP-meglumine antimoniate nanocomposite reduces *Leishmania amazonensis* infection in macrophages

**DOI:** 10.1186/s12866-021-02267-2

**Published:** 2021-07-12

**Authors:** Ana Patricia Cacua Gélvez, José Antonio Picanço Diniz Junior, Rebecca Thereza Silva Santa Brígida, Ana Paula Drummond Rodrigues

**Affiliations:** 1 Evandro Chagas Institute, Secretary of Health Surveillance, Laboratory of Electron Microscopy, Ministry of Health, Av. Almirante Barroso, 492, Marco, Pará 66090-000 Belém, Brazil; 2 Postgraduate Program in Biology of Infectious and Parasitic Agents, Federal University of Pará, R. Augusto Corrêa, 01 - Guamá, Pará CEP: 66075-110 Belém, Brazil

**Keywords:** AgNP-PVP-MA nanocomposite, Cytokine production, Antileishmanial, Ultrastructural alterations, Leishmaniasis

## Abstract

**Background:**

Leishmaniasis is an infectious disease caused by parasites of the genus *Leishmania* and presents different clinical manifestations. The adverse effects, immunosuppression and resistant strains associated with this disease necessitate the development of new drugs. Nanoparticles have shown potential as alternative antileishmanial drugs. We showed in a previous study the biosynthesis, characterization and ideal concentration of a nanocomposite that promoted leishmanicidal activity. In the present study, we conducted a specific analysis to show the mechanism of action of AgNP-PVP-MA (silver nanoparticle–polyvinylpyrrolidone-[meglumine antimoniate (Glucantime®)]) nanocomposite during *Leishmania amazonensis* infection *in vitro*.

**Results:**

Through ultrastructural analysis, we observed significant alterations, such as the presence of small vesicles in the flagellar pocket and in the extracellular membrane, myelin-like structure formation in the Golgi complex and mitochondria, flagellum and plasma membrane rupture, and electrodense material deposition at the edges of the parasite nucleus in both evolutive forms. Furthermore, the *Leishmania* parasite infection index in macrophages decreased significantly after treatment, and nitric oxide and reactive oxygen species production levels were determined. Additionally, inflammatory, and pro-inflammatory cytokine and chemokine production levels were evaluated. The IL-4, TNF-α and MIP-1α levels increased significantly, while the IL-17 A level decreased significantly after treatment.

**Conclusions:**

Thus, we demonstrate in this study that the AgNP-PVP-MA nanocomposite has leishmanial potential, and the mechanism of action was demonstrated for the first time, showing that this bioproduct seems to be a potential alternative treatment for leishmaniasis.

## Background

Leishmaniasis is a parasitic disease and has various clinical manifestations: cutaneous, mucocutaneous and visceral. The complex interactions between the infectious capacity of different species of *Leishmania* and the immune status of the human host are related to different clinical forms of leishmaniasis; the disease represents a global public health problem [1]. It is estimated that approximately 350 million people live in regions where there is a risk of acquiring infection; 1.3 million new cases are recorded and approximately 20,000 to 30,000 deaths occur each year [1].

In Brazil, American cutaneous leishmaniasis (ACL) caused by parasites of the genus *Leishmania* is transmitted by sandflies of the genus *Lutzomyia* [2], and the treatment comprises first-line drugs such as pentavalent antimonials, which, in Brazil, are represented by the antimoniate of N-methylglucamine or meglumine antimoniate [3]. In specific cases or due to a lack of response to antimonial treatment, second-line drugs such as amphotericin B (AMB) and pentamidine are used [4]. Due to the resistance of some *Leishmania* species to the drugs mentioned above, suppressed immunity and different scenarios of adverse effects in patients when treated with them [5], the search for new treatment criteria and therapeutic alternatives with reduced side effects that improve the effectiveness of traditional methods are studied continuously [6]. Therefore, it is noteworthy that the delivery of therapeutic compounds to specific targets has been one of the problems in the treatment of diseases and that the conventional administration of some drugs is characterized by limited efficacy, low distribution and poor target selectivity [6, 7].

In recent years, the tools offered by nanotechnology techniques have been applied to the development of new medicines through the handling of materials and/or nanocomposites due to their electrical, optical, magnetic and physicochemical properties, making them beneficial for medical, physical, optical and electronic applications [7]. With the advent of nanodrug technology, delivery systems based on liposomes, nanoemulsions, micelles and dendrimers conjugated to polymeric nanoparticles, solid lipid nanoparticles, metal nanoparticles, carbon nanotubes and drugs of interest (meglumine antimoniate, amphotericin and miltefosine, among others) [8, 9] have shown excellent potential, with antileishmanial activity against *Leishmania major* [10], *Leishmania infantum* [11], *Leishmania tropica* [12], and *Leishmania amazonensis* [13].

The interaction of nanoparticles with current drugs used in the treatment of leishmaniasis is of great interest for three reasons: 1- dosages currently used to treat leishmaniasis are well known; 2- they have the potential to treat resistant strains; and 3- better therapeutic results could be achieved [6, 7]. Thus, conjugation between compounds has generated positive results in several studies for the control of leishmaniasis, mainly leading to an increased inhibitory action of conjugated drugs against parasites compared to that of the individual drug treatments [12]. Our research group first biosynthesized, optimized, characterized, and demonstrated that the nanocomposite AgNP-PVP-MA reduced, *in vitro*, the viability of promastigotes and the macrophage infection of amastigotes, without causing cytotoxic effects on mouse macrophages, *in vitro*. However, we do not know how the AgNP-PVP-MA reacts or acts in the different evolutive forms of *Leishmania amazonensis*, or even if the nanocomposite could act indirectly to induce an immune response during the macrophage infection. Posteriorly, some additional questions arose from the answers.

## Materials and methods preparation of the nanocomposite

The nanocomposite is a combination of AgNP (100 % stock solution of AgNP) obtained by biological synthesis from *Aspergillus flavus* culture (size < 10 nm) mixed with the polymer PVP (10 %) obtained the AgNP-PVP. After that, the stock solution of Glucantime (1 mg/mL) was added in the AgNP-PVP solution to obtained the nanocomposite AgNP-PVP-MA with 50 µg/mL as final concentration of MA. To define the appropriate concentration used in the present study, we performed several preliminary biological tests. The process of nanocomposite production and the determination of the ideal concentration for use were detailed in our previous publication [13]. All experiments were performed in accordance with the arrive guidelines.

### Biological tests

#### Definition of nanocomposite concentration and reference drug

During the optimization of the nanocomposite AgNP-PVP-MA in previous published research, AgNP, PVP and MA, individually and together, as well as the reference drug amphotericin B were tested at different concentrations, both in macrophage cells and in the promastigote and amastigote forms of the *Leishmania amazonensis* parasite. Based on these results, the appropriate concentrations were defined, considering the affecting viability of the parasite and the reduced or absent cytotoxicity in macrophages (data not shown). In our preliminary evaluation, as the inhibitory effect in the amastigote forms was lower when the Glucantime® was used as reference drug, we consider the amphotericin B more reliable as a reference drug for comparisons to the nanocomposite effect (data not shown).

#### Parasites

Promastigote forms of *Leishmania amazonensis* (MHO/BR/M26361) were obtained in Novy-MacNeal-Nicolle (NNN) medium from the Evandro Chagas Institute Leishmaniasis Program and maintained in RPMI 1640 medium (Sigma-Aldrich®, USA) supplemented with 10 % fetal bovine serum (FBS - Gibco® Thermo Fisher Scientific, USA) in biological oxygen demand (BOD) at 27 °C.

#### In vitro macrophage test

Intraperitoneal macrophages (IPΦ) were obtained from 8- to 10-week-old BALB/c albino mice. The animals were produced and obtained from the Animal Facility of Evandro Chagas Institute. The animals were euthanized for cell collection by washing the peritoneal cavity, according to the Commission on the Ethics of Animal Experiments of the Evandro Chagas Institute. The aspirated material was concentrated by centrifugation for 10 min at 2500 *g* and at 10 °C. After that, the cells were counted in a Neubauer chamber, and the concentration was adjusted to 2 × 10^5^/mL. Macrophages were transferred to 24-well culture plates or 75 cm^2^ cell culture flasks incubated at 37 °C in a humidified atmosphere containing 5 % CO_2_ for 1 h for cell adhesion. After this period, cells were washed with sterile phosphate-buffered saline (PBS), pH 7.2, to remove nonadherent cells, and Dulbecco’s modified Eagle’s medium (DMEM - Sigma-Aldrich®, USA), pH 7.2, with penicillin/streptomycin and supplemented with 10 % FBS. Cells were maintained at 37 °C in 5 % CO_2_ atmosphere for 24 h (h).

#### Anti-amastigote activity

To determine the infection rate in macrophages, these cells were cultured as specified in the previous section on cover slips on the bottom of the culture plate and allowed to interact with *Leishmania amazonensis* promastigotes in a ratio of 1:10 for 3 h at 35 °C and an atmosphere of 5 % CO_2_. Then, the supernatant was discarded, and DMEM with 10 % FBS was added and incubated for 24 h under the conditions mentioned. Subsequently, infected cells were treated for 24 h with AgNP-PVP-MA nanocomposite with the concentration already described, under the same temperature and atmosphere conditions as those described above. As a positive control, amphotericin B was used at a concentration of 0.5 *µ*g/mL, and infected cells without treatment were used as a negative control. After the treatment period, the supernatants were collected and stored in microtubes in a *freezer* (-70 °C) for further nitrite and cytokine evaluations. Subsequently, the coverslips were washed with PBS and fixed with 3 % formaldehyde for 30 min. Cells were incubated for 10 min with DAPI (Molecular Probes Invitrogen®, USA) for nuclei detection (1:100) and with Alexa Fluor® 594 phalloidin (Molecular Probes Invitrogen®, USA) for actin filaments (1:200) and were subsequently mounted on glass slides using ProLong Gold® Antifade reagent (Molecular Probes Invitrogen®, USA). Noninfected and infected cells were counted in random fields using an Axio Scope A1, Carl Zeiss Microscopy, using ZENLite software. At least 100 infected macrophages were counted, and the number of parasites was determined by examining three coverslips for each treatment. The results are expressed as an infectivity index (II) according to Eq. (1) [13, 14]:

1 $$$$Infectivity\;Index\;\left(II\right)=\frac{\%\;Infected\;macrophages\;\times\;Parasites\;internalized/Cell}{Total\;number\;of\;macrophages}$$

#### Ultrastructural analysis of *Leishmania amazonensis*

Promastigote forms of *Leishmania amazonensis* were cultivated in culture bottles at a parasite concentration of 1 × 10^6^/mL and treated for 96 h, while intracellular cultures were treated for 24 h; both developmental forms of the parasite were treated with AgNP-PVP-MA nanocomposite in the concentration and conditions described above. After the treatment period, the samples were processed for evaluation by scanning electron microscopy (SEM) and transmission electron microscopy (TEM). The samples were fixed for one hour at room temperature in a solution containing 2.5 % glutaraldehyde, 4 % formaldehyde and 2.5 % sucrose in Phem buffer (composed of 0.235 g of MgCl_2_, 2.61 g of KCl, 1.9 g of EGTA, 2.6 g of HEPES, and 10.4 g of PIPES in 250 ml of Milli-Q water at pH 7.2). Subsequently, the cells were washed in 0.1 M cacodylate buffer and incubated for 1 h at room temperature in a solution containing 1 % osmium tetroxide and 0.8 % potassium ferrocyanide. Then, for SEM, dehydration was performed in an increasing series of ethanol concentrations for 10 min, and samples were dried at the critical point (Modelo K 850 – Mark EMITCH) using CO_2_. The samples were fixed in an appropriate support (*stub*) using carbon tape and metalized (Metallizer Emitech K 550 – England) with a gold film approximately 2 nm thick for further evaluation with a Zeiss LEO 1450VP SEM. For TEM analyses, cells were fixed and post-fixed as described above, stained with 1 % uranyl, and dehydrated in an increasing series of acetone concentrations for 10 min. After dehydration, cells were slowly impregnated with Epon resin at concentrations of 2:1, 1:1, 1:2 (acetone: Epon) and finally pure Epon for 12 h. Subsequently, the material was incubated in pure Epon + DMP30 for 6 h, followed by polymerization at 60 °C for 48 h. The polymerized blocks were cut by ultramicrotomy and contrasted with lead citrate for 3 min for further evaluation with a Zeiss EM 900 TEM.

#### Nitrite quantitation

Nitric oxide production was estimated by determining the concentration of nitrite (NO^−^ _2_) present in the supernatant of the macrophage cultures infected by stationary phase promastigotes after treatment with the AgNP-PVP-MA nanocomposite at the concentration described above for 24 h using the Griess method [15]. The culture supernatant was collected, and nitrite production was evaluated using a Griess Reagent Kit (Molecular Probes Invitrogen®, USA) for nitrite quantitation according to the methods described [16] and to the manufacturer´s instructions. The nitrite concentration was determined using dilutions of sodium nitrite (NaNO_2_) to create a standard curve, and the results were expressed in micromolar (*µ*M).

#### Measurement of reactive oxygen species levels

Macrophage cultures (5 × 10^5^/mL) infected with *Leishmania amazonensis* (5 × 10^6^/mL) under the same conditions as described above were treated or untreated with AgNP-PVP-MA nanocomposite in the concentration described above for 24 h. After that, the cells were washed with PBS, pH 7.2, and subsequently incubated with 5.0 *µ*M CellROX® Green reagents (Molecular Probes Invitrogen®, USA) in PBS for oxidative stress detection, according to the manufacturer´s instructions. After a 30-min incubation in the dark at 37 °C and 5 % CO_2_, the cells were washed with PBS, pH 7.2, and fixed in 3.7–4.0 % paraformaldehyde for 15 min, followed by washing in PBS. Cells without AgNP-PVP-MA nanocomposites were used as a negative control. Fluorescence was measured using a VICTOR Multilabel Plate Reader X fluorometer at 485 nm and 520 nm for excitation and emission, respectively. The results were expressed as optical density (OD).

#### Detection and quantification of cytokines and chemokines

Cytokines present in the supernatants of *Leishmania*-infected cultures after infection and treatment for 24 h with AgNP-PVP-MA nanocomposite in the concentration already described were quantified by flow cytometry. The cytokines IL-2, IL-4, IL-6, IFN-γ, IL-10, IL-17 A and TNF-α were measured using BD Cytometric Bead Array (CBA) – Cytokine Kit Mouse Th1/Th2/Th17; the cytokine IL-1β, GM-CSF and the chemokines MCP-1, MIP-1α and RANTES were measured using BD CBA Mouse Flex Set according to the manufacturer´s instructions. The data were obtained with BD FACSCanto II using FACSDiva software (BD Biosciences, USA), and analysis was performed in FCAP Array 3.0. The results were expressed as pg/mL and were calculated according to a standard curve.

#### Statistical analysis

All assays were performed in triplicate in three different experiments. The results are expressed with statistical significance determined by analysis of variance followed by Student’s t-test. A *p*-value ≤ 0.05 was considered significant. The data were analyzed using GraphPad Prism 7.

## Results

### Anti-amastigote activity promoted by the AgNP-PVP-MA nanocomposite

**Fig. 1:**
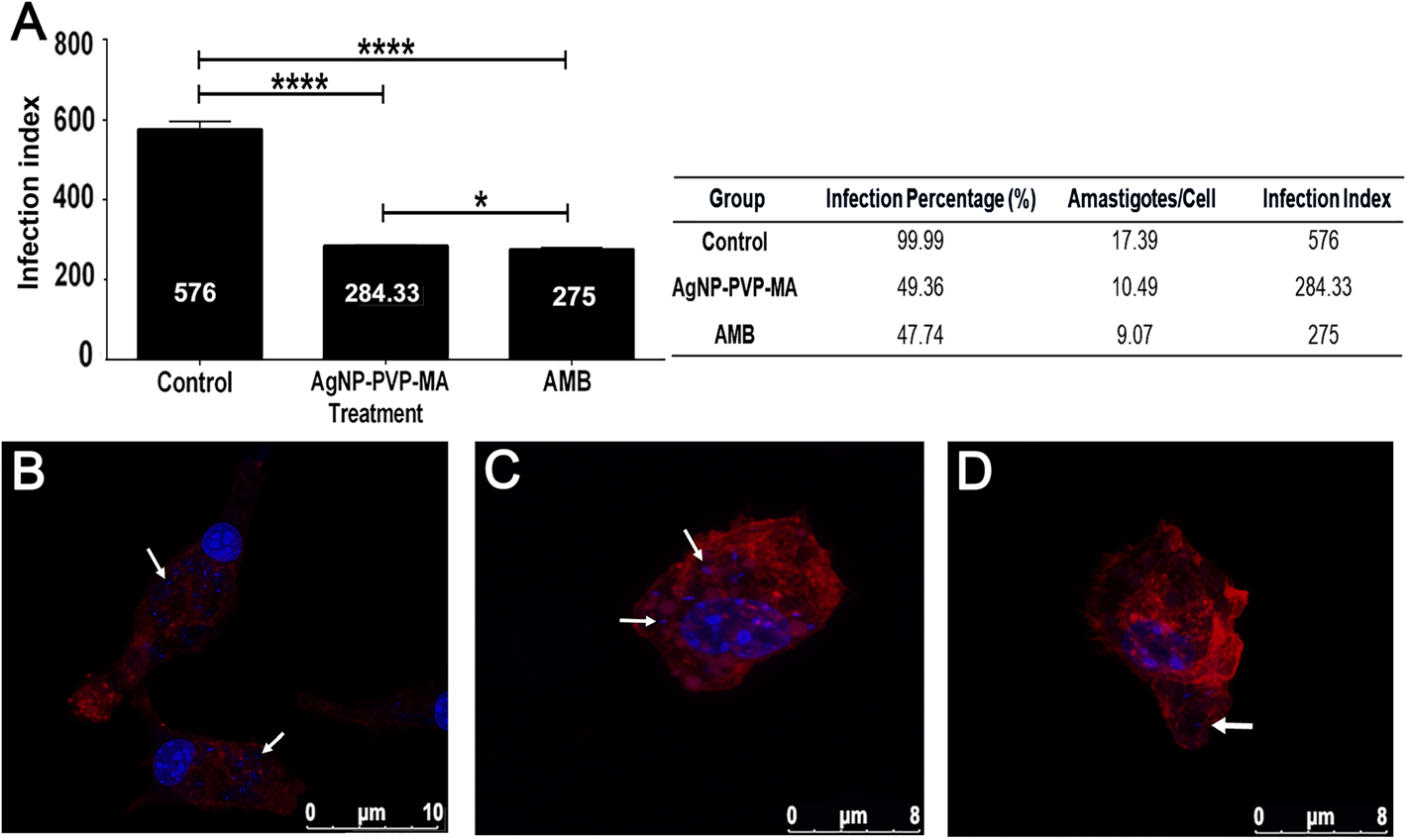
Infection index of macrophages infected with *Leishmania amazonensis*. Graph showing the levels of infection index obtained after study and descriptive table of mean values for infection percentage, number of amastigotes per macrophage and infection index (**A**). Fluorescence labeling of infected macrophages, untreated (negative control); after 24 h of incubation (**B**). Fluorescence labeling of infected macrophages treated with AgNP-PVP-MA nanocomposite (AgNP to 1 %, 0.1 g of PVP and 50 µg/mL of MA) incubated for 24 h. (**C**). Fluorescence labeling of infected macrophages treated with AMB (positive control) at a concentration of 0.5 *µ*g/mL incubated for 24 h. Actin filaments - phalloidin in red (1:200) and DAPI in blue (1:100) was used (**D**). White arrow: amastigote forms. Control group: macrophages infected but untreated. AgNP-PVP-MA group: macrophages infected and treated with the nanocomposite. AMB group: macrophages infected and treated with amphotericin B. Analysis of variance, Student’s t-test: *p ≤ 0.05; **** *p* ≤ 0.0001

Following treatment with AgNP-PVP-MA nanocomposite in the concentration described or amphotericin B (0.5 *µ*g/mL), as a reference drug, after 24 h of incubation, infection levels were similarly reduced with both the reference drug (275 mean value of infection) and the nanocomposite (284.33 mean value of infection) compared to those in the untreated control group (Fig. 1A), with 576 mean value of infection, demonstrating the anti-leishmanial potential of the nanocomposite. Additionally, a descriptive table were presented with the mean values ​​of the infection percentage, number of amastigotes per macrophage and infection index (Fig. 1A). A significant reduction in the amastigote number in the group treated with AgNP-PVP-MA nanocomposite (Fig. 1C – white arrow) compared to those in the untreated control group (Fig. 1B) and the amphotericin B-treated group (Fig. 1D – white arrow) was determined by fluorescence microscopy.

### Morphological alterations in promastigotes and amastigotes promoted by the AgNP-PVP-MA nanocomposite

**Fig. 2:**
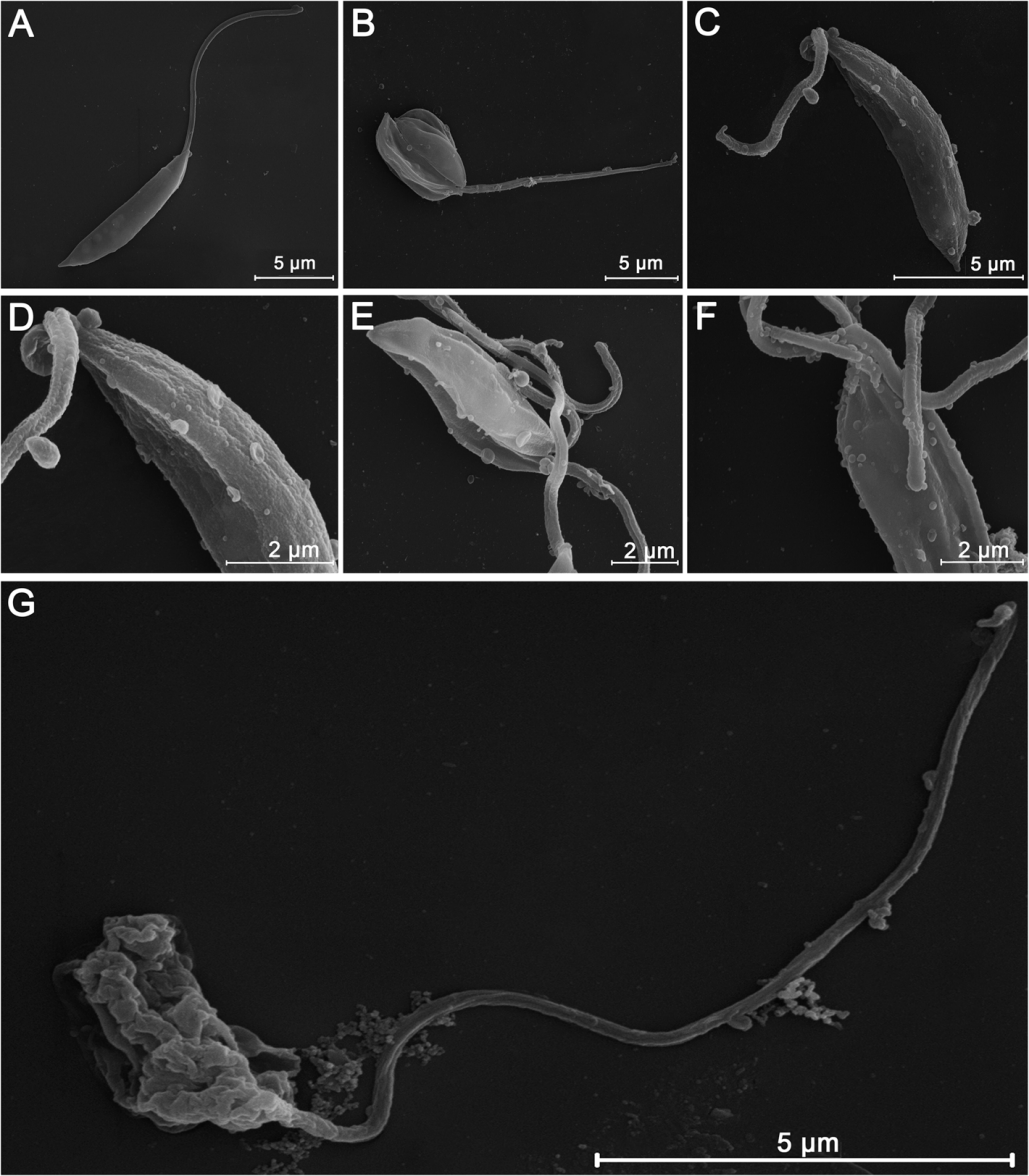
Scanning electron micrographs of *Leishmania amazonensis* promastigotes treated with the AgNP-PVP-MA nanocomposite (AgNP to 1 %, 0.1 g of PVP and 50 µg/mL of MA) for 96 h. A promastigote without treatment had preserved typical elongated morphology with externalized flagellum in excellent conditions (**A**). A promastigote with the presence of changes in body size (rounded body), compared with promastigotes in the untreated group, generated by the action of the AgNP-PVP-MA nanocomposite (**B**). A promastigote treated with AgNP-PVP-MA and presenting changes in body and the flagellum size, with rough type structure (**C**). Treated promastigote showing changes in the structure of the parasite, such as a wrinkled cell membrane (**D**). Promastigotes presenting alterations in the body shape with the presence of small vesicles (EVs) in the body (**E**). The presence of small vesicles (EVs) in both the body and flagellum after treatment (**F**). A promastigote altered by the effect of AMB (positive control) at a concentration of 0.5 *µ*g/mL, with extensive membrane destruction (**G**)

First, using SEM, several alterations in promastigote morphology were observed, including changes in body shape, wrinkled cell membrane, rounded body and shortened flagellum (Fig. 2B, C and D), compared with that of promastigotes in the untreated group (Fig. 2A). In addition, were observed small vesicles (SVs) in the body and flagellum (Fig. 2D-F). The group treated with amphotericin B (Fig. 2G) presented a body morphology completely altered by the drug effects, with extensive membrane destruction, despite it presented an elongated flagellum.

**Fig. 3:**
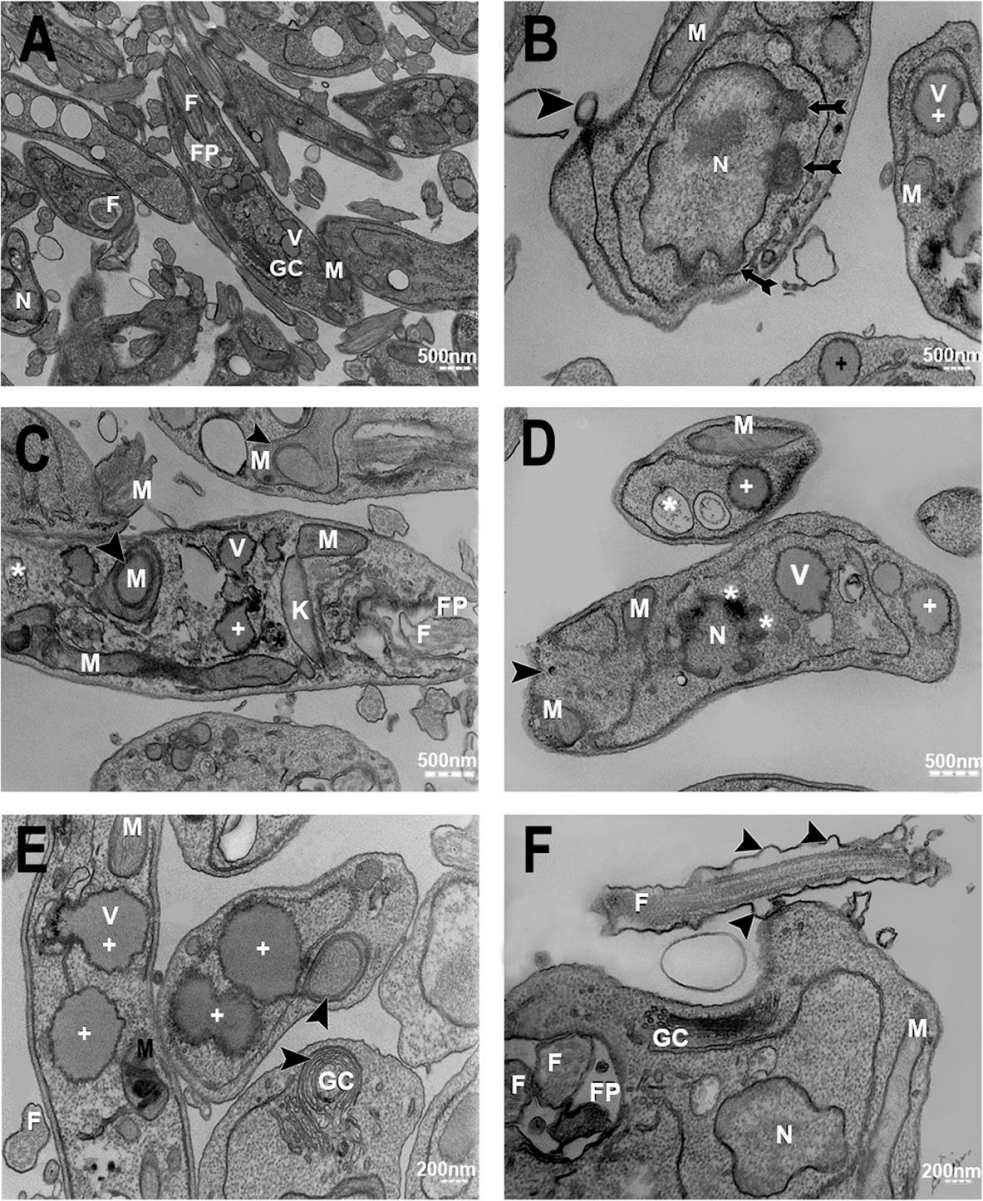
Transmission electron micrographs of *Leishmania amazonensis* promastigotes after treatment with the AgNP-PVP-MA nanocomposite (AgNP to 1 %, 0.1 g of PVP and 50 µg/mL of MA) for 96 h. Promastigote forms without treatment (negative control), with typical morphology (**A**). Promastigotes displaying chromatin condensation in the nuclear periphery [arrows] (**B**). Changes in parasite vesicles [+], with possible accumulation of the nanocomposite [*], and the presence of myelin-like structures in mitochondria [►] (**C**). Plasma membrane rupture [►] and the presence of vesicles containing electrondense material [asterisks] at the edges of the nucleus [N] (**D**). Altered vesicles [+] and the presence of myelin-like structures in the Golgi complex [►] (**E**). Parasite flagellar membrane changes [►] (**F**). **N**: Nucleus. **M**: Mitochondrion. **F**: Flagellum. **FP**: Flagellar pocket. **GC**: Golgi complex. **V**: Vesicles. **K**: Kinetoplast

After evaluating the surface structure of the parasite by SEM, ultrastructural changes in the organelles of the promastigote and amastigote forms of *Leishmania amazonensis* were determined after AgNP-PVP-MA nanocomposite treatment by TEM. Promastigotes without treatment had typical organelle morphology (Fig. 3A); however, when exposed to the nanocomposite, the parasite displayed vesicles containing electron-dense material in the nuclear membranes (Fig. 3B - arrows), showing the beginning of the apoptotic process, with chromatin condensation in the nuclear periphery (Fig. 3D - asterisks). In addition, a myelin-like structure was observed in mitochondria (Fig. 3C - arrowhead) and the Golgi complex (Fig. 3E - arrowhead), suggesting the accumulation of the nanocomposites in vesicles (Fig. 3C – asterisks, and, 3E - crosses). Plasma membrane rupture was also observed (Fig. 3D - arrowhead), in addition to the presence of morphological alterations in the flagellar membrane (Fig. 3 F - arrowhead).

**Fig. 4:**
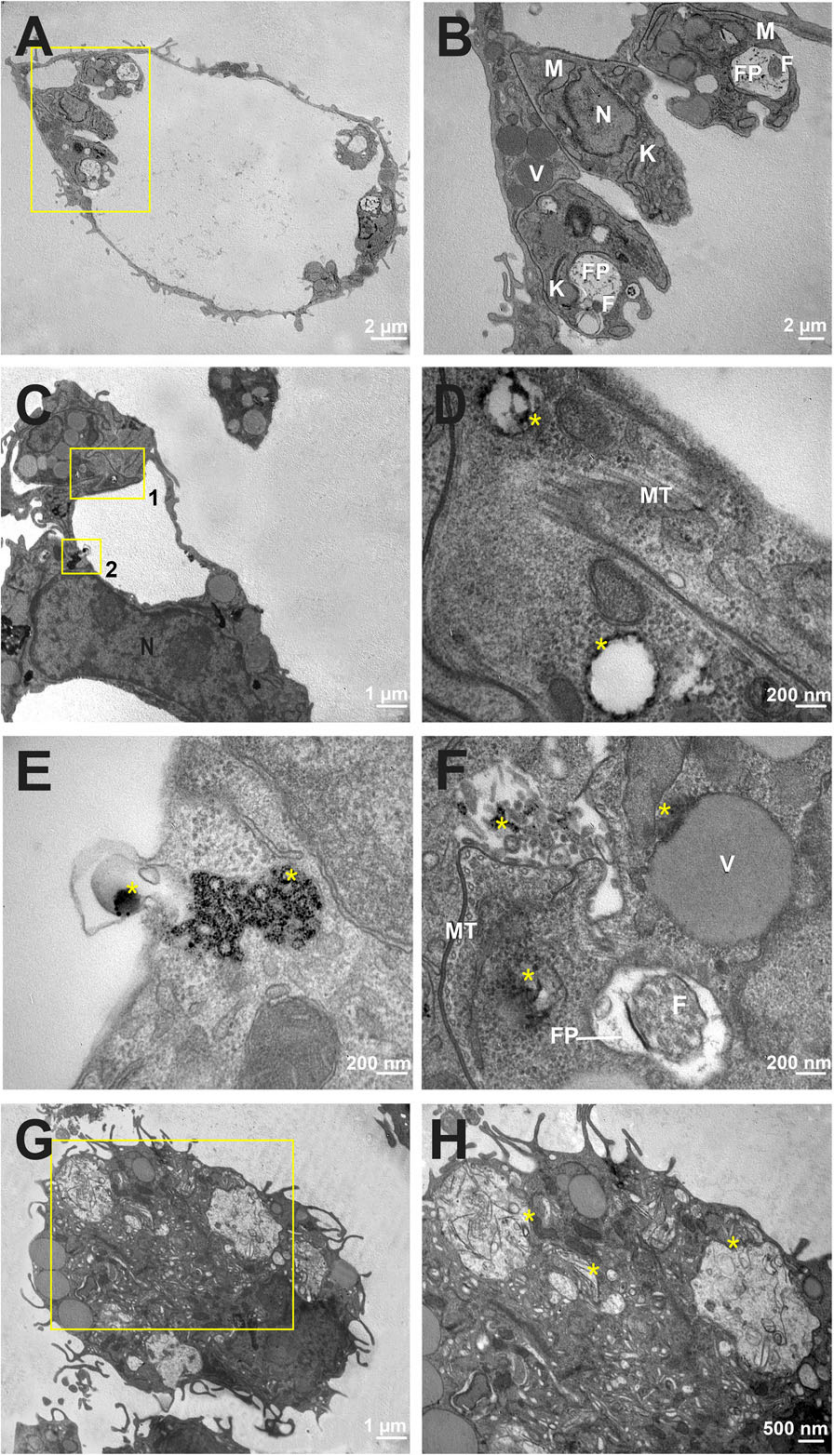
Transmission electron micrographs of *Leishmania amazonensis* intracellular amastigotes after treatment with the AgNP-PVP-MA nanocomposite (AgNP to 1 %, 0.1 g of PVP and 50 µg/mL of MA) for 24 h. The image shows the parasitophorous vacuole of an infected macrophage in control group (**A**). Amplification of image (A), showing amastigotes with typical morphology (**B**). A macrophage infected and treated with AgNP-PVP-MA, showing the presence of the nanocomposite in some regions [selected insets 1 and 2] (**C**). Amplification of inset 1 [image (C)]; presence of AgNP-PVP-MA (asterisks) in vesicles and close to the subpellicular microtubule was observed (**D**). Amplification of inset 2 [image (C)], with the presence of the nanocomposite (asterisks) inside the parasitophorous vacuole (**E**). The nanocomposite (asterisks) near the flagellar pocket, multivesicular bodies and close to the subpellicular microtubule (**F**). A macrophage infected with *Leishmania amazonensis* and treated with the reference drug AMB (positive control) at a concentration of 0.5 *µ*g/mL, showing the absence of amastigotes [inset] (**G**). Amplification of the inset [image (G)], showing the presence of cellular debris inside the parasitophorous vacuole [asterisks] (**H**). **N**: Nucleus. **M**: Mitochondrion. **F**: Flagellum. **FP**: Flagellar pocket. **MT**: Subpellicular microtubule

The preservation of organelles and typical morphology were observed in the infected and untreated macrophages, as in the negative control (Fig. 4A and B). The presence of AgNP-PVP-MA nanocomposite was visualized in different areas in *Leishmania amazonensis* infected and treated macrophages (Fig. 4C – inset). The presence of the nanocomposite was observed in some regions: parasitophorous vacuole (Fig. 4E - asterisks), inside the parasite vesicles (Fig. 4D), and in proximity to the subpellicular microtubule (Fig. 4D F), flagellar pocket and multivesicular bodies (Fig. 4F - asterisk). The positive control group macrophages infected and treated with AMB showed the absence of amastigote forms (Fig. 4G - inset) and several incidences of cellular debris inside the parasitophorous vacuoles (Fig. 4H - asterisks).

### ROS and NO levels in infected macrophages treated with AgNP-PVP-MA nanocomposite

**Fig. 5:**
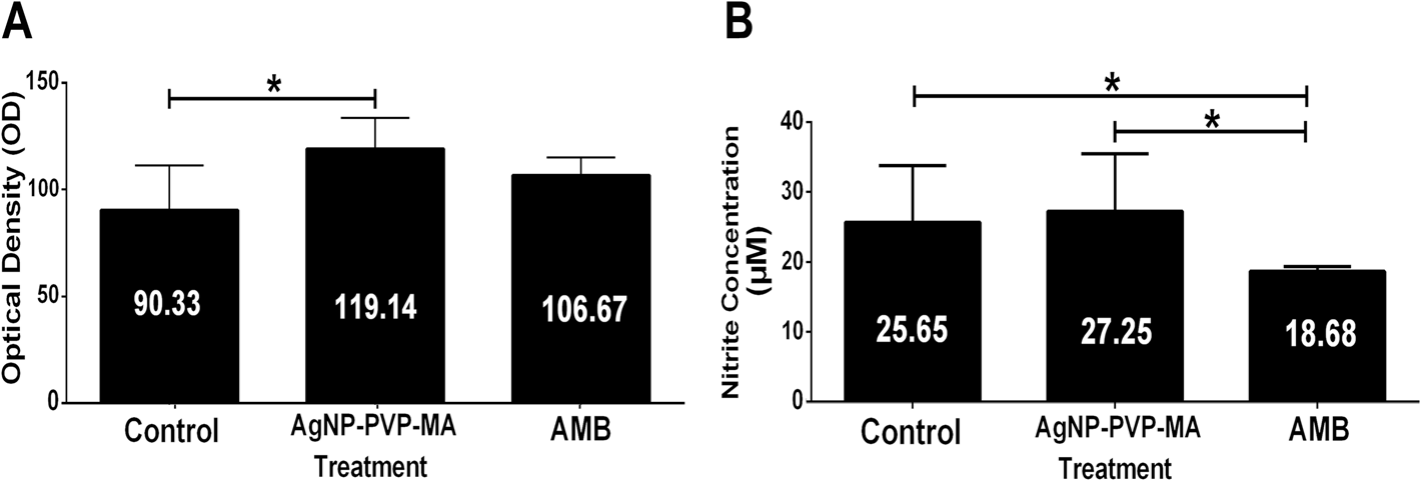
Determination of reactive oxygen species levels and nitrite production after treatment with AgNP-PVP-MA nanocomposite (AgNP to 1 %, 0.1 g of PVP and 50 µg/mL of MA). Generation of ROS in macrophages infected with *Leishmania amazonensis* and treated with nanocomposite for 24 h (**A**). Levels of nitrite production in macrophages infected with *Leishmania amazonensis* and treated with nanocomposite for 24 h (**B**). Control group: macrophages infected and untreated. AgNP-PVP-MA group: macrophages infected and treated with the nanocomposite. AMB group: macrophages infected and treated with amphotericin B at a concentration of 0.5 *µ*g/mL. The results were expressed as mean ± standard deviation of triplicate sample (*n* = 3) in three different experiments with analysis of variance, Student’s t-test; *p ≤ 0.05

After the treatment of infected macrophages, differences in NO production and ROS were observed between experimental groups and the untreated control group (infected macrophages without treatment). In the ROS measurement, the highest levels were observed in the group treated with the AgNP-PVP-MA nanocomposite, with significant differences compared to untreated control group (Fig. 5A). Regarding indirect NO detection, no significant differences were found between the untreated control group and the group treated with the nanocomposite, but there were significant differences in relation to the control group with the amphotericin B drug (Fig. 5B).

### Production of cytokines and chemokines in infected macrophages treated with AgNP-PVP-MA nanocomposite

**Fig. 6:**
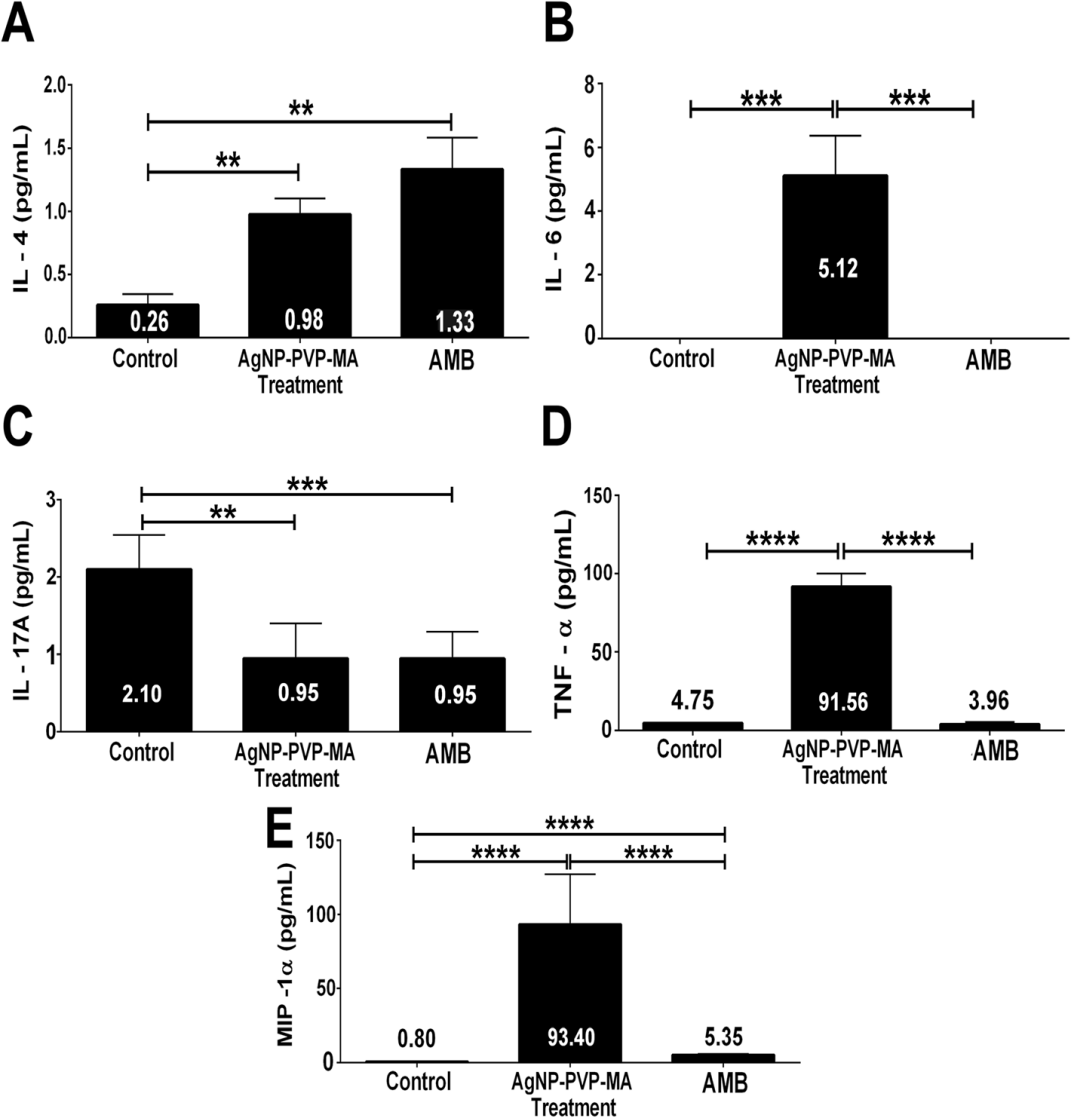
Cytokine and chemokine levels after treatment with AgNP-PVP-MA nanocomposite (AgNP to 1 %, 0.1 g of PVP and 50 µg/mL of MA) for 24 h. Levels of interleukin 4 [IL-4] (**A**). Levels of tumor necrosis factor alpha [TNF-α] (**B**). Levels of interleukin 17 A [IL-17 A] (**C**). Levels of macrophage inflammatory protein-1 alpha [MIP-1α] (**D**). Control group: macrophages infected and untreated. AgNP-PVP-MA group: macrophages infected and treated with the nanocomposite. AMB group: macrophages infected and treated with amphotericin B at a concentration of 0.5 µg/mL. The results were expressed as mean ± standard deviation of triplicate sample (*n* = 3) in three different experiments with analysis of variance, Student’s t-test; *p ≤ 0.05; **p ≤ 0.005; ***p ≤ 0.0005; ****p ≤ 0.0001

From the infected and treated macrophage culture supernatants, the production levels of cytokines and chemokine profiles were determined. First, IL-4, and TNF-α levels increased after treatment compared to those in the control group (untreated cells). IL-4 showed substantial production in the groups treated with AgNP-PVP-MA (approximately 3-fold increased) or AMB (approximately 5-fold increased) compared to the IL-4 production in the control group (Fig. 6A). TNF-α (Fig. 6B) and the chemokine MIP-1α (Fig. 6D) were produced in larger quantities in the nanocomposite-treated group than in both the untreated and AMB groups. Conversely, although IL-17 A was produced in all experimental groups, we observed reductions in IL-17 A levels in the treated groups [AgNP-PVP-MA and AMB] (Fig. 6C). The data of cytokines IL-2, IFN-γ, IL-6, IL-10, IL-1β, and GM-CSF and the chemokines MCP-1 and RANTES were not shown because their level of detection was below the detection limit or were not detected during this assay.

## Discussion

The conjugation of compounds, such as drugs and nanoparticles, is a nanotechnological option in the generation of new nanoformulations and/or drugs, with specific targeting [6, 7, 17] due to increased therapeutic efficacy, inhibitory potential against pathogens and reduced toxicity in patients [18, 19]. Recently, our group developed the new AgNP-PVP-MA nanocomposite as a potential alternative for leishmaniasis treatment [13]; however, the mechanism of action in relation to ultrastructural alterations in the parasite and the immune response of the infected host cell have not been determined. Thus, the purpose of this study was to complement the previous approach to determine the possible pathways and responses promoted by the nanocomposite.

Cell morphology is characterized by presenting unique alterations, dependent on the type of cell death induced in response to various stimuli [20], where through electron microscopy it is possible to elucidate the mechanism of action of drugs [21, 22]. Therefore, through SEM and TEM, ultrastructural alterations of the parasite were evaluated to establish the possible type of cell death (i.e., necrosis, apoptosis or autophagy). In the SEM analysis of promastigotes treated with the nanocomposite, a shortening of the flagellum was observed, as well as changes in body shape with rough type structure and rounded body, and the presence of small extracellular vesicles (EVs) in the parasite membrane, including the flagellar membrane, when compared to the untreated control group. Moreover, rupture of the plasma and flagellar membranes was observed, with the presence of EVs close to them, revealing another possible path of cell death, associated with the response of external stimuli, in different biological and pathological processes [23, 24]. In addition, EVs are usually present in all biological fluids and are considered possible biomarkers for infectious diseases [25–27], such as those caused by *Toxoplasma gondii* [28], *Leishmania major* [29], and *Plasmodium yoelii* [30]. However, the content of the EVs and their role in the pathogenesis and immunological evasion are not yet well defined for the pathogenic species [31]. It is known that the secretion of EVs is associated with the transmission of stress signals [26], affecting the motility, regulation, and growth of the parasite [32], with strong pro-inflammatory properties [33] stimulated by the accumulation of Ca^+^ in the cytoplasm [34, 35]. We believe that the nanocomposite could act in this way of Ca^+^ metabolism to release the EVs; however, further assays are necessary to prove this hypothesis.

Another structural alteration observed was the induction of vesicles containing electron-dense material in the periphery of the nucleus, similar to those observed in the beginning of the apoptotic process, with chromatin condensation. The findings observed in the present study, such as nucleus alterations, rupture of the cell membrane and damage in mitochondria, were observed in the same species of parasites treated with different nanoparticles and amiodarone drugs [36, 37].

A myelin-like structure was observed in the treated groups in both the mitochondria and Golgi complex of parasites, essential organelles involved in the energy metabolism, oxidative phosphorylation, cell secretion and the distribution of macromolecules necessary for parasite survival. The myelin-like structure and the alterations in the ROS levels are associated with the autophagy process [38–40], and in our study, the nanocomposite seems to alter this pathway of death. The AgNP has a preference for some organelles such as mitochondria and nucleus, generating a potential collapse of the mitochondrial membrane when interacting with membrane proteins [41, 42], inducing cytotoxicity through numerous mechanisms, including oxidative stress [43], favoring the production of ROS, and the release of Ag^+^ ions by dissolving AgNP. This behavior was observed [36, 44] when evaluating the *Leishmania amazonensis* parasite with silver nanoparticles, with observations of apoptosis-like events, a reduced percentage of infected macrophages, ROS production, loss of mitochondrial integrity and damage to the promastigote and amastigote forms of the membrane. The production of ROS in our study was not high when compared to the stimulus generated by the individual AgNP, possibly due to the types of interactions present in the nanocomposite and the presence of other compounds. However, oxidative stress is an important mechanism that is associated with cytotoxicity, and the AgNP present in our nanocomposite seems to favor the production of ROS in a controlled manner, as demonstrated by [36, 43] and [45], culminating in an intracellular parasite load reduction. Therefore, knowledge of how ROS influence the activation of the cell death program is important for revealing mechanisms that can be used for therapeutic intervention in major human diseases [39]. In general, AgNPs are expected to induce toxicity through ROS induction, which is dependent on the nanoparticle type, chemistry and structure [46]. Thus, in our study, we observed that this mechanism is selective, as we could observe the presence of nanocomposites inside of the parasite and inside the parasitophorous vacuoles, showing that the delivery system works well, promoting a direct activity in intracellular amastigotes, increasing the ROS production of infected macrophages with reduction in the parasite number and without affecting the morphology of the host cell. These findings allow an understanding of how the cellular uptake of nanocomposite works inside the host cell and/or parasite, as those mechanisms are detected using several comprehensive technological tools, such as electron microscopy, helping to elucidate the pathways involved in the nanoformulation carrier and possible organelle targets [47].

Another important point determined in this study was the cytokine and chemokine production after the expose of *Leishmania*-infected cells to the nanocomposite. Successful immunity to *Leishmania* depends on the recruitment of appropriate immune effector cells: macrophages, NK cells, CD4^+^ and CD8^+^ cells, and Th1, Th2, Treg and Th17 cell differentiation [48], where anti-inflammatory and pro-inflammatory cytokines and chemokines play relevant roles in the regulation of cell differentiation, recruiting resident and migratory cells involved in the resistance and susceptibility to and the immunopathogenesis of *Leishmania* infection [49, 50]. Studies have reported that the resistance to leishmaniasis infection is related to a Th1 response, with the production of mainly pro-inflammatory cytokines, such as IL-12, which is indispensable in the production of IFN-γ (not detected in our study), and, synergistically with TNF-α, activates inducible nitric oxide synthase (iNOS or NOS2) to produce nitric oxide (NO), an important molecule for the reduction of intracellular parasites [48, 51]. The cytokines IL-1 and IL-2 (not detected in our study) are responsible for the activation of macrophages and neutrophils as well as the production of ROS [49, 52, 53]. In contrast, some parasites, such as *Leishmania amazonensis*, guarantee their survival by modulating the host immune system, promoting a host susceptibility associated with a Th2 response [54], with the production of IL-4, IL-5, IL-10 and IL-13, responsible for parasite replication and persistence [51, 55, 56].

In our study, only the cytokines IL-4, IL-17 A, and TNF-α and the chemokine MIP-1α were detected. It has been reported that TNF-α and IL-6 are important in the formation of the apoptotic process [51, 57], which seemed to occur in our study once suggestive alterations in the morphology of the parasite were observed, as discussed above. Additionally, studies have reported that AgNP and AuNP alone increase the secretion of pro-inflammatory cytokines such as IL-1ß, IL-2, IL-6, TNF-α and prostaglandin E2 (PGE-2), which are involved in parasite elimination mechanisms [58, 59], and the MA are able to induce the production of pro-inflammatory cytokines, which increases the phagocytic capacity of monocytes, macrophages and neutrophils [60, 61].

The presence of AgNP and MA in our nanocomposite seems to be related to the higher production of the pro-inflammatory cytokine TNF-α (compared with control groups) and chemokine MIP-1-α, seems to induce the inflammatory process, usually observed in the early stages of infection and could be related with the reduction of the parasite load through the production of ROS which is also an inflammatory marker. Besides, the chemokine MIP-1α (CCL3) is known to induce IL-1, IL-6, and TNF-α production with chemotactic and pro-inflammatory effects and acts as homeostasis promoter culminating in a Th1 response in the presence of receptors CCL3 and CCR5, fundamental in the control of leishmaniasis [50, 51].

Thus, the Th1 response is fundamental in the control of leishmaniasis and is responsible for the activation of macrophages in skin lesions, along with CCL2, reducing the parasitic load [50, 51, 62]. Moreover, the nanocomposite seems to induce this kind of response in *Leishmania*-infected cells. The other chemokines evaluated, GM-CSF and MCP-1(CCL2), which have important roles in early immunity against cutaneous leishmaniasis [63], were not detected in our study, possibly due to the absence of some cytokines and chemokines associated with their expression, which were not promoted by the nanocomposite, at least in the first 24 h, demonstrating other ways to fight the parasite.

Conversely, IL-17 A, responsible for activating signaling pathways that lead to chemokine induction with TNF-α and IL-1, inducing the production of IL-6, GM-CSF, IL-1β, TGF-β, IL-8 and MCP-1 [57], but was little detected in our study, indicating the activation of other routes different of Th17 responses.

It is well known that macrophages are one of the first cell types to act as a defense against *Leishmania* parasites during the infectious process, and in addition to ROS, they produce NO and cytokines and chemokines as important mechanisms to control leishmaniasis disease [49]. This control mechanism depends on the species of *Leishmania* involved in clinical manifestations, the different immunological pathways in which pro- and anti-inflammatory cytokines play different roles in resistance or susceptibility, and the immunopathogenesis of the host [48, 64]. For *Leishmania amazonensis*, one of the main etiological agents of leishmaniasis in the Amazonian region [64], a Th2 response against infection is generally developed, confirmed by the presence of high levels of IL-4 and IL-10, and accompanied by the production of IgG1 antibody isotypes [54, 56]. However, some strains of *Leishmania amazonensis* guarantee their survival by modulating the host immune system, inducing immunosuppression and are able to reduce the inflammatory response, especially in the initial stages of infection process, which decreases the effectiveness of treatment [50, 54, 64]. Cytokines and chemokines have complex and important roles in the promotion and suppression of immune cells in the leishmaniasis infectious process. The IL-4 is usually associated with a Th2 response, responsible for the replication and persistence of the *Leishmania* parasite. However, recently it was demonstrated the dual role of this cytokines, as anti-Leishmania in the presence of MIF, GM-CSF, TNF-α, IFN-γ, IL-2, IL-7, IL-15, and, pro-Leishmania in the presence of IL-10, TGF-β, IL-6, IL-13, MIF, IL-3; showing its diverse interventions in the infectious process of Leishmaniasis [65]. Despite in our study we observed higher production of this cytokines in the nanocomposite group compared with control group, the production was lower and was not able to suppress the TNF production.

In this way, we believe that the nanocomposite seems to partially reverse this inhibitory mechanism generated by the *Leishmania amazonensis* and, associated with the production of ROS and expression of Th1 cytokines and chemokines, culminates in a reduction of intracellular amastigotes, as well as significant ultrastructural alteration, showing the selective action of this nanocomposite against the *Leishmania* parasite.

Thus, these findings were important for providing a better understanding of the possible mechanism of action of the AgNP-PVP-MA nanocomposite as a promising alternative option for the control of leishmaniasis. In this way, the next step for the application of the nanocomposite is the incorporation of it in a non-invasive vehicle that could be used as local dermal treatment for cutaneous leishmaniasis.

## Conclusions

**Fig. 7:**
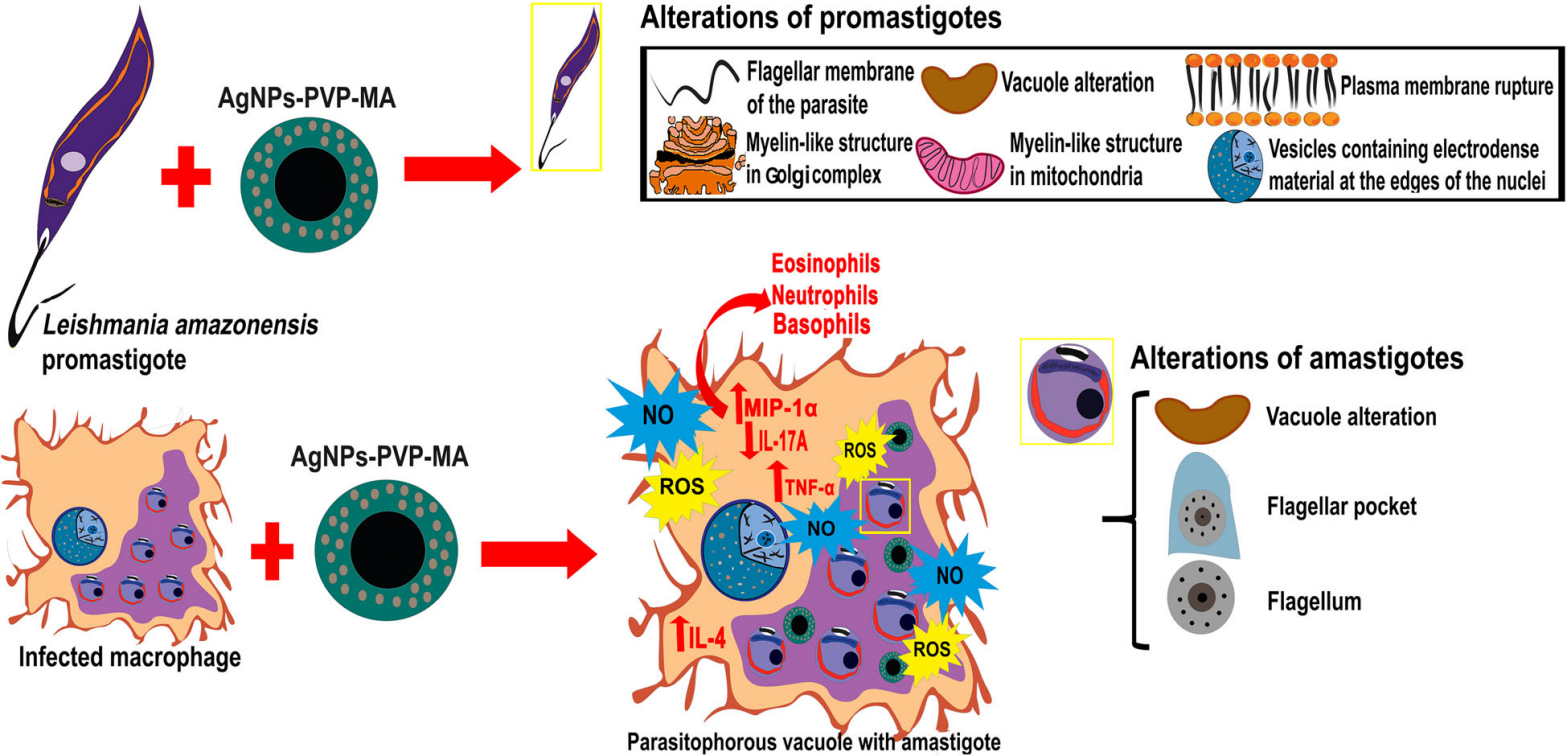
Cell behavior after treatment with AgNP-PVP-MA nanocomposite. After infection of macrophages with *Leishmania amazonensis* and later treatment with the AgNP-PVP-MA, generated that, as mechanism of action promoted by nanocomposite, the pro-inflammatory and anti-inflammatory cytokines TNF-α, IL-4, and IL-17 A and the chemokine MIP-1α were expressed, as the main mediators during infection of the parasite this study; being involved in different roles as resistance, susceptibility and immunopathogenesis, and being indispensable during infection control of parasite in the leishmaniasis. Resistance to leishmaniasis is related to the development at a Th1 response and its respective production of pro-inflammatory cytokines, like TNF-α, IL-6, and, IL-17 A, which are responsible for activating macrophages and generating the death of parasites by the production of NO and ROS, responsible for the elimination of intracellular amastigotes. Our results may indicate that the ROS and NO pathways are not stimulated by the nanocomposite, since their expression was low in ROS and NO, which leads to a low activation and stress of macrophages in the presence of the nanocomposite, can being other pathways can being involved in the action mechanism these. On the other hand, less IL-4 and IL-17 A were produced and IL-17 was reduced in the presence of the nanocomposite. In addition, the chemokine MIP-1α produced in large quantities by macrophages in our study is characterized by chemotactic and pro-inflammatory effects and is crucial for immune responses toward infection and inflammation. Furthermore, neutrophils, eosinophils and basophils, are favored by the presence of TNF-α, responsible for pro-inflammatory functions as well as the death of parasites because of their cytotoxic function. They also induce the synthesis and release of other pro-inflammatory cytokines by macrophages. Parasite death was determined by the presence of ultrastructural changes in the two developmental forms of the *Leishmania*, which were highlighted in the promastigote form as a compromised nucleus, possible initiation of the apoptotic process and chromatin condensation in the nuclear periphery and the presence of a myelin-like structure in the mitochondrial and Golgi complex organelles. In the case of the amastigote form, the specificity of the AgNP-PVP-MA nanocomposite was predominant by ultrastructural alterations in the flagellum, vacuole and flagellar pocket, with presence of nanocomposite in vesicles and close to the subpellicular microtubule, showing promoted possibly an mechanism of action directed by specific parasite organelles of *Leishmania* and its potential as a new treatment alternative for cutaneous leishmaniasis disease with the stimulation of a Th1 response. ROS: reactive oxygen species. NO: nitric oxide. TNF-α: tumor necrosis factor alpha. IL-4: Interleukin 4. IL-6: Interleukin 6. IL-17 A: Interleukin 17 A. MIP-1α: macrophage inflammatory protein-1 alpha. iNOS_2_: inducible nitric oxide synthase 2. AgNP: silver nanoparticles

The important findings generated from the present study allowed to establish, for the first time, the effect of the AgNP-PVP-MA nanocomposite, in the parasite and infected host cell, reversing the inhibitory process of the immune response generated by the species of *Leishmania amazonensis*, stimulated a Th1 response and generated ultrastructural alterations in relevant organelles in the two forms of the parasite, activating an important cell death mechanism by the autophagy process, elucidated by electron microscopy (summarized in Fig. 7). In this way, the present study showed that AgNP-PVP-MA acts in the different evolutive forms of *Leishmania amazonensis* by a direct activity or indirect response through activation of macrophages response.

## Data Availability

All data generated or analysed during this study are included in this published article.

## Abbreviations

MA: Meglumine Antimoniate (also known Glucantime®)
AgNP: Silver Nanoparticles
PVP: Polyvinylpyrrolidone
NO: Nitric Oxide
NO−2: Nitrite
NaNO2: Sodium Nitrite
ROS: Reactive Oxygen Species
CBA: Cytometric Bead Array
AMB: Amphotericin B
N: Nucleus
M: Mitochondria
F: Flagellum
FP: Flagellar Pocket
GC: Golgi Complex
V: Vacuole
K: Kinetoplast
MT: Subpellicular Microtubules
MIP-1α: Macrophage Inflammatory Protein-1 Alpha
EVs: Extracellular Vesicles
iNOS2: Inducible Nitric Oxide Synthase 2
ACL: American Cutaneous Leishmaniasis
CONCEA: National Council for the Control of Animal Experimentation
CEUA: Commission on the Ethics of Animal Experiments
IPΦ: Intraperitoneal Macrophages
